# Involvement of NRF2 in Breast Cancer and Possible Therapeutical Role of Polyphenols and Melatonin

**DOI:** 10.3390/molecules26071853

**Published:** 2021-03-25

**Authors:** Alev Tascioglu Aliyev, Emiliano Panieri, Višnja Stepanić, Hande Gurer-Orhan, Luciano Saso

**Affiliations:** 1 Department of Toxicology, Faculty of Pharmacy, Ege University, 35040 Izmir, Turkey; alev.aliyev@ibg.edu.tr; 2 Department of Physiology and Pharmacology “Vittorio Erspamer”, Sapienza University of Rome, P. le Aldo Moro 5, 00185 Rome, Italy; emiliano.panieri@hotmail.it (E.P.); luciano.saso@uniroma1.it (L.S.); 3 Laboratory for Machine Learning and Knowledge Representation, Ruđer Bošković Institute, Bijenička 54, 10000 Zagreb, Croatia; Visnja.Stepanic@irb.hr

**Keywords:** breast cancer, NRF2, oxidative stress, polyphenols, melatonin

## Abstract

Oxidative stress is defined as a disturbance in the prooxidant/antioxidant balance in favor of the former and a loss of control over redox signaling processes, leading to potential biomolecular damage. It is involved in the etiology of many diseases, varying from diabetes to neurodegenerative diseases and cancer. Nuclear factor erythroid 2-related factor 2 (NRF2) is a transcription factor and reported as one of the most important oxidative stress regulators. Due to its regulatory role in the expression of numerous cytoprotective genes involved in the antioxidant and anti-inflammatory responses, the modulation of NRF2 seems to be a promising approach in the prevention and treatment of cancer. Breast cancer is the prevalent type of tumor in women and is the leading cause of death among female cancers. Oxidative stress-related mechanisms are known to be involved in breast cancer, and therefore, NRF2 is considered to be beneficial in its prevention. However, its overactivation may lead to a negative clinical impact on breast cancer therapy by causing chemoresistance. Some known “oxidative stress modulators”, such as melatonin and polyphenols, are suggested to play an important role in the prevention and treatment of cancer, where the activation of NRF2 is reported as a possible underlying mechanism. In the present review, the potential involvement of oxidative stress and NRF2 in breast cancer will be reviewed, and the role of the NRF2 modulators—namely, polyphenols and melatonin—in the treatment of breast cancer will be discussed.

## 1. Introduction

According to the global cancer statistics data (GLOBOCAN 2020) from the International Agency for Research on Cancer, breast cancer has the highest incidence and mortality rates among women [1]. The mortality-to-incidence ratio in breast cancer has been decreasing over the years, which indicates that the survival rate is gradually increasing. However, our century still has the economical, physical and psychological burden of cancer therapy. Despite the remarkable advances in scientific knowledge, the risk factors and the mechanisms responsible for breast cancer are still under investigation.

Breast cancer is a heterogeneous disease and is classified as a luminal A (estrogen receptor (ER) and/or progesterone receptor (PR) (+) and human epidermal growth factor receptor 2 (HER2) (−), with low ki-67 levels); luminal B (ER and/or PR (+) and HER2 (−), with high ki-67 levels); basal-like (also called triple-negative due to the lack of ER, PR and HER2 expression) and HER2-enriched (ER and PR (−) and HER (+)) molecular subtypes [2]. The hormone receptor status is not the only prognostic marker for the disease, but other molecular markers such as the PD-L1 gene presence [3], tissue localization (ductal and lobular) and invasion status of the cancer cells are also used to define better treatment strategies. With a better understanding of the genetic and epigenetic alterations and estrogen and oxidative stress signaling pathways, breast cancer therapy is expected to take a decisive step towards the personalized and effective treatments of patients. 

## 2. Mechanisms Underlying Breast Cancer

Hanahan and Weinberg defined the “hallmarks of cancer” to understand tumoral changes in normal cells for all cancer types. To explain the tumoral behavior of the cell, the authors addressed eight key features: unrestrained proliferation, avoided growth suppressor signaling, resistance to cell death, sustained replication, induction of angiogenesis, activation of invasion and metastasis and altered cellular metabolism, including redox homeostasis and evaded immune destruction [4,5]. More generally, the stages of carcinogenesis can be summarized as a multistep process wherein excessive and irreversible cellular DNA damage initiates cancer cell formation, while the subsequent alteration of the signaling pathways promotes the sustained proliferation and genetic instability of preneoplastic cells, leading to their clonal expansion. Afterwards, the occurrence of further genetic changes converts these cells into cancer cells that progressively acquire more aggressive features, culminating in tissue invasion and metastasis formation [6,7].

Multiple risk factors, such as aging, early menarche/late menopause, hormone replacement therapy, oral contraceptive usage, obesity, family history, benign lesions, radiation therapy, nutrition and other lifestyle habits, are involved in breast cancer development [8]. Estrogens are a driven factor for the promotion and progression of around 75–85% of breast cancer patients, which have hormone receptor-positive subtypes (luminal A and luminal B). However, patients with basal-like (triple-negative) breast cancer have inherited breast cancer gene (BRCA) mutations. The current section explains the risk factors and risk factor-related underlying mechanisms of breast cancer, with a particular emphasis on oxidative stress.

### 2.1. Oxidative Stress-Independent Mechanisms

Family history, race and ethnicity are related to the development of breast cancer. Inherited genetic mutations—in other words, germline mutations in specific genes—were found to be related to breast cancer development. At the mid-1990s, the role of BRCA1 and BRCA2 mutations in elevating breast cancer risk was considered [9]. A prospective study with 3886 breast cancer patients showed that a cumulative risk was 72% for BRCA1 and 69% for BRCA2 carriers [10]. The genetic analyses of patients revealed that tumor-suppressor genes—namely, PTEN, p53, STK11 and cell adhesion regulator CDH1 genes, are high-penetrance breast cancer-susceptibility genes and increase the breast cancer risk more than fourfold. Mutations in moderate-penetrance genes such as PALB2, ATM and CHEK2 were also reported to increase the risk of breast cancer occurrence two to four times [11].

Other conditions—specifically, hormone replacement therapy, early menarche/late menopause, a history of never being pregnant and/or breastfeeding and oral contraceptive usage—are stated as risk factors for the development of breast cancer, since elevated estrogen exposure was found to enhance the aberrant epithelial cell proliferation and to cause neoplastic formation [12]. Hilton et al., in a seminal paper, reviewed the relationship between steroid hormone receptors and breast cancer [13]. The authors reported an aberrant hormonal activation in steroid receptor-positive breast cancer results with a metabolic switch between autocrine signaling, which led to cell proliferation and prevented proapoptotic signaling. Estrogens and progesterone also regulate histone modifications, and enhanced levels of those hormones can cause epigenetic changes [13].

Nutritional and lifestyle habits such as the consumption of alcohol, high-calorie diets, obesity and physical inactivity contribute to breast cancer initiation and/or promotion [14]. For example, alcohol consumption increases the steroid hormone levels, which, in turn, alters the DNA methylation status by affecting one carbon metabolism [15]. A high-calorie intake with reduced physical activity led to an increase in the body mass index (BMI). Being overweight (25 ≤ BMI < 30 kg/m^2^) and obese (BMI ≥ 30 kg/m^2^) was found to be significantly associated with breast cancer progression in postmenopausal women [16]. Mechanistically, adipose cells secrete two adipokines, antioncogenic adiponectin and pro-oncogenic leptin. Jarde et al. revealed that the leptin expression was higher than adiponectin expression in epithelial ductal breast cancers, while, in the normal tissue adjacent to cancer, the expression of adiponectin was prevalent [17]. Mechanistically, obesity-related alterations of adipose stem cells increase leptin secretion, which becomes a source for enhanced estrogen-related tumor growth by upregulating the estrogen receptor and aromatase [18].

An age and cancer incidence correlation was well-established, although the underlying mechanisms are still under investigation. Oxidative stress-independent age-related mechanisms were reviewed, such as accumulated somatic mutations, epigenetic changes in DNA methylation and chromatin remodeling and changes in breast tissue and the cell microenvironment [19].

### 2.2. Oxidative Stress-Dependent Mechanisms

Oxidative stress was defined as “a disturbance in prooxidant-antioxidant balance in favor of the former” by Sies at 1985 [20], while Dean Jones further expanded this definition, including also the disruption of redox signaling circuitries [21]. The production of reactive oxygen species (ROS) and reactive nitrogen species (RNS) is a normal consequence of the cell metabolism. Mitochondria produce ATP through oxidative phosphorylation, involving the passage of electrons through a series of complexes, part of the electron transport chain, promoting the reduction of molecular oxygen to water. During this process, electrons can leak through the inner membrane space and produce superoxide radical ion, which is the precursor of various ROS and RNS molecules. Cells such as macrophages, leukocytes and monocytes also produce superoxide radical anion (O_2_^●—^) via NADPH oxidase activity. On the other hand, mammalian cells maintain the redox balance through the coordinated activity of antioxidant enzymes and antioxidant molecules. For example, the superoxide dismutase (SOD) converts O_2_^●—^ to the less electrophilic hydrogen peroxide H_2_O_2_, which is rapidly detoxified into water H_2_O and oxygen molecules O_2_ by glutathione peroxidase (GPx), catalase (CAT) or peroxiredoxin (Prx). However, the excessive production of ROS and/or inadequate supply of antioxidant molecules (e.g., endogenous glutathione) may cause a further reaction between H_2_O_2_ and Fe^2+^ ions (Fenton reaction) and produce highly reactive hydroxyl radical ^●^OH. Due to its electrophilic properties, the hydroxyl radical attacks nucleophilic cellular molecules such as proteins, lipids and DNA. In such a way, increased oxidative stress generates DNA adducts, protein damage and lipid peroxidation products such as the 4-hydroxynonenal (4-HNE) [6]. DNA adduct formation is mainly initiated by ^●^OH. Hydroxyl radical attacks the 2′-deoxyribose of purine and pyrimidine bases. As a consequence, this reaction generates 8-hydroxy-2’-deoxyguanosine (8-OH-dG) and 8-oxo-7,8-dihydro-2’-deoxyguanosine (8-oxo-dG), two well-recognized markers of oxidative stress-related DNA adduct formation [22]. Insufficient DNA repair leads to the accumulation of DNA adducts in the cells. Moreover, the division of damaged cells promotes tumoral changes [23]. As a result, oxidative stress derived by different sources such as ionizing radiation, exposure to xenobiotics or their metabolites, aging, alcohol consumption, obesity and estrogens may trigger carcinogenesis.

Nour Eldin et al. showed that the plasmatic levels of 8-OH-dG exhibit a steady-state increase from normal breast tissue to benign and malignant lesions for different patient groups [24]. In the same study, the authors also showed that malignant breast tumors with highly invasive subtypes were also characterized by higher 8-OH-dG levels than tumors with less invasive behaviors. In contrast, Sova et al. did not find a positive association between the tumor stages and serum 8-OH-dG levels, while, conversely, the content of 8-OH-dG was found to be related with the tumor stage in the immunohistochemical analyses [25]. By comparing the serum levels and the immunohistochemical content of 8-OH-dG, the study revealed that both these features were strongly associated with tumor aggressiveness. Taken together, it can be concluded that breast cancer development partially occurs via DNA adduct formation by oxidative stress, and the disease progression may be monitored by the quantitative analysis of oxidative stress-related DNA products.

#### ROS Formation via Estrogen Metabolism

Breast cancer development by estrogens can derive from two different mechanisms: estrogen receptor activation and the alteration of estrogen metabolism-related pathways [12,26].

Generally, chemicals are metabolized through phase I reactions that include oxidation, reduction and hydroxylation of the substance and phase II reactions that promote enzymatic conjugation of the metabolite with endogenous molecules, such as glutathione (GSH), sulfate and glucuronic acid, and facilitate its elimination. Similarly, estrogens (estradiol and estrone) undergo phase I metabolism by cytochrome p-450 enzymes and are converted to 2-hydroxycatechol estrogen or to 4-hydroxycatechol metabolites. Catechol metabolites of estrogens are detoxified mainly by catechol-O-methyl transferase via conjugation. At the same time, catechol estrogens undergo autooxidation, which promotes the formation of a hydroxyl radical. Catechol metabolites can also be oxidized to highly reactive estrogen 3,4-semiquinone, estradiol-2,3-semiquinone and, additionally, to estrogen 3,4-quinone and estradiol-2,3-quinone. Commonly, the phase II detoxification of quinone metabolites are catalyzed by glutathione-S-transferase [12,27]. However, under certain circumstances, such as excessive exposure to estrogens by hormone replacement therapy, oral contraceptive usage and/or limited detoxification capacity, reactive estrogen metabolites can react with DNA and cause genomic instability, as extensively reviewed by Cavalieri et al. [27].

### 2.3. Role of Nuclear Factor Erythroid 2-Related Factor 2 (NRF2) in Breast Cancer

**Figure 1:**
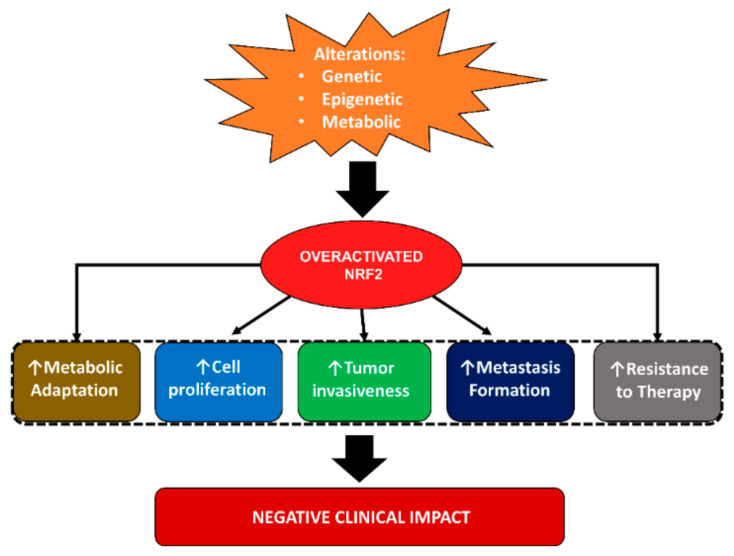
Roles of nuclear factor erythroid 2-related factor 2 (NRF2) in breast cancer.

Compelling evidence from the last decade indicates that nuclear factor erythroid 2-related factor 2 (NRF2) is a master regulator of a cytoprotective response centered on the activation of detoxifying mechanisms in response to oxidative/electrophilic stress or xenobiotics. Importantly, it is well-recognized that sustained NRF2 signaling in cancer cells can be instrumental to the orchestration of a pro-oncogenic “program” that ultimately promotes the malignant progression and the development of therapy resistance, leading to poor clinical outcomes. An increased NRF2 expression in breast cancer patients resulted in a lower overall survival and disease-free survival [28]. Despite that NRF2 can participate in the regulation of oncogenic signaling and cancer-specific hallmarks, other data suggest that its activation in normal cells can exert a chemopreventive role by suppressing ROS-dependent DNA damage and carcinogenesis. In agreement with the still-unresolved scientific debate, this reflects the dual role of NRF2 in cancer and suggests that both pro-oncogenic and antioncogenic activities can be exerted by this transcription factor, depending on additional factors that require a case-by-case assessment (Figure 1). In the following section, we will describe in more detail the role of NRF2 in the regulation of breast cancer hallmarks.

#### 2.3.1. NRF2 in Breast Cancer Cell Proliferation, Growth, Invasion and Metastasis

In a quite recent study, the genetic silencing of NRF2 was found to significantly impair cell proliferation and migration in the MDA-MB-231 and MCF-7 breast cancer cells by downregulating the small GTPase and transforming protein RhoA, while its reconstituted expression was able to promote an increased growth rate and invasiveness, also restoring the levels of RhoA. Mechanistically, NRF2 was shown to bind the promoter region of ERR1 (nuclear member receptor estrogen-related receptor α), a protein that promotes RhoA ubiquitination, suggesting that the transcriptional repression of ERR1 might prevent RhoA degradation and, therefore, facilitate the activation of its downstream pro-oncogenic signaling [29]. A positive role for NRF2 in controlling the proliferation of breast cancer cells was also described in a study wherein an mir-101 mimic significantly reduced the mRNA levels of NRF2, impairing both the proliferation and colony formation rate of MCF-7 cells, while opposite changes were produced by mir-101 inhibition [30]. Along similar lines of evidence, De Blasio and colleagues reported that NRF2 enhanced both the proliferation and antioxidant capacity of triple-negative MDA-MB-231 breast cancer cells through the downmodulation of miR-29b-1-5p expression [31]. In marked contrast, however, other studies support the notion that decreased NRF2 signaling, rather than its overactivation, can promote cancer growth and cell proliferation. In this regard, Xu et al. demonstrated that the genetic silencing of NRF2 in MCF-7 breast cancer cells resistant to Adriamycin was paralleled by a marked upregulation of the CDCA4 protein (cell division cycle-associated protein 4) that was ultimately responsible of the enhanced growth and proliferation of these cells in vitro [32]. Similarly, by using MDA-MB-231 breast cancer cells, NRF2 was recently shown to induce the expression of ferroportin (FPN), a transmembrane protein that regulates the intracellular iron content by promoting iron efflux [33]. Here, the genetic silencing of FPN accelerated MDA-MB-231 cell proliferation and growth in vitro and in vivo, while its forced expression produced the opposite changes. Of note, a further analysis revealed that the NRF2 mRNA and protein levels were significantly lower in tumor specimens from breast cancer patients compared to the corresponding adjacent tissues and paralleled by similar changes in the FPN content. The authors concluded that defective NRF2 signaling can induce alterations in the iron metabolism that might ultimately promote breast cancer growth. Taken together, these studies suggest that NRF2 might exert divergent effects on breast cancer cell proliferation and growth, most likely depending on the specific context and genetic background of its activation or repression.

Despite that sustained NRF2 activation is considered a driver of the malignant progression of several types of tumors, its role in breast cancer (migration, invasion and metastasis) still remains a matter of intense debate due to conflicting data. In this regard, the initial studies identified NRF2 as a negative regulator of RON (Recepteur d’ origine nantais) gene expression, a tyrosine kinase receptor frequently overactivated in breast tumors and associated with tamoxifen resistance, as well as metastatic disease [34]. Here, from the comparative immunohistochemical analysis of the tissue microarray samples, a high NRF2 and low RON expression was observed in normal tissues, while a low or absent NRF2 content and high RON levels were found in breast tumors. A mechanistic investigation conducted on a panel of breast cancer cells (and other types of tumors) revealed that NRF2 was able to directly bind to the RON promoter and repress its genetic induction, while, accordingly, the reconstituted expression of NRF2 not only decreased the RON levels but, also, impaired breast cancer cell migration and invasion. Along similar lines of evidence, other research conducted on 4T1 and JC murine breast cancer cells has shown that the flavonoid fisetin, a known NRF2 activator, can exert a tumor-suppressive function by decreasing the expression of matrix metalloproteinases (MMP-2 and MMP-9), two key regulators of tumor invasion and metastatic spreading [35]. Mechanistically, fisetin was found to enhance the NRF2 nuclear accumulation and heme oxygenase-1 (HO-1) expression, causing a significant decrease in the mRNA levels and the activity of MMP-2 and MMP-9, while these changes were antagonized by NRF2 silencing. Therefore, the authors proposed that the NRF2-dependent activation of HO-1 can attenuate the metastatic potential of breast cancer cells by inhibiting the MMP-2 and MMP-9 expression and enzymatic activity but, also, cell motility through the NRF2–HO-1 axis, in agreement with the evidence from other types of tumors [36]. Additionally, a very recent study investigated the role of NRF2 in the transcriptional regulation of the chemokine CXCL13 and its receptor CXCR5, two known drivers of breast cancer cells migration and metastatic dissemination, from primary breast tumors to lymph nodes [37]. Here, NRF2 was found to directly recognize multiple sites within the CXCL13 gene promoter and to negatively modulate its transcription when overexpressed in MDA-MB-231 cells, also suppressing the activating effects normally induced by the transcription factor RelA. The authors concluded that NRF2 is a negative regulator of CXCL13 and a potential tumor suppressor in breast cancer, as also suggested by the presence of higher NRF2 levels in ER (−) breast cancer cells and the increased frequency of CXCL13/CXCR5 co-expression in ER (+) breast cancer cells with lower NRF2 contents. In a marked contrast, however, a number of recent studies have indicated that NRF2 plays a pro-oncogenic role in breast cancer, promoting tumor invasion and metastasis spreading. For instance, Zhou et al. showed that the oncoprotein HBXIP (mammalian hepatitis B X-interacting protein) plays a critical role in modulating cancer malignancy and tumor progression and is involved in breast carcinoma progression [38]. More specifically, an immunohistochemistry assay, qPCR on the mRNA and immunofluorescence on the protein levels confirmed that HBXIP expression was positively correlated with NRF2 expression in clinical samples from breast cancer tissues, suggesting their implication in breast cancer development. The mechanistic insights on MCF-7 breast cancer cells showed that HBXIP reduced the ROS levels by promoting nuclear NRF2 accumulation and the subsequent transactivation of NRF2-dependent target genes such as NAD(P)H dehydrogenase-1 (NQO1), glutamate-cysteine ligase catalytic and modifier subunits (GCLC and GCLM) and AKR1C1. Of note, HBXIP silencing attenuated the expression of NRF2 and its nuclear accumulation markedly enhancing the intracellular ROS levels. Furthermore, by comparing the impact of HBXIP-induced NRF2 activation in MDA-MB-436 cells with the stable knockdown or reconstituted expression of HBXIP, the authors revealed that the proliferation, migration and invasion abilities of these cells were strongly impaired by the genetic depletion or site-specific mutation of HBXIP, while, conversely, were rescued by the re-expression of the HBXIP-wt form. These observations were further confirmed in vivo, since HBXIP knockdown led to reduced nodule formation in mouse lungs and impaired tumor growth in metastatic and orthotopic xenograft models. Lastly, both these phenotypic changes were reverted by HBXIP-wt reconstitution. In summary, the authors concluded that HBXIP promotes the malignancy of breast cancer by modulating abnormal redox regulation in vitro and in vivo through the overactivation of NRF2 signaling.

#### 2.3.2. NRF2 in the Regulation of Breast Cancer Cell Stemness and Therapy Resistance

In another work, the group of Mi-Kyoung investigated the role of NRF2 in the modulation of the cancer stem cell (CSC) phenotype, establishing a breast CSC-like model by isolating the subpopulations of MCF-7 (doxorubicin sensitive), MCF-7/ADR (doxorubicin-resistant) and MDA-MB-231 cells with high expression levels from cluster of differentiation 44 (CD44 high), a common CSC marker associated with drug resistance, tumor recurrence and metastasis formation [39]. Notably, a detailed analysis revealed that the expression of CD44, along with typical CSC markers such as SOX2, OCT-4 and MDR-1, was significantly higher in MCF7/ADR than in MCF7 cells and that NRF2 signaling was also more active in CD44 high cells compared to the CD44 low counterparts. Mechanistic studies on the stable CD44 high cell line (ADR44P) revealed that the increased NRF2 content was caused by CD44-p62 signaling, while the genetic silencing of NRF2 was able to markedly suppress the aggressive phenotype of CSC, including drug resistance, colony/sphere formation and cell migration in vitro, but, also, tumor growth in vivo [40]. Therefore, this indicates that the CD44–NRF2 axis might be an effective therapeutic target to impair the stress resistance and survival of the CD44 high CSC population in breast cancer, in agreement with the previous findings.

Regarding the clinical impact of NRF2, a consistent body of evidence supports the notion that its activation is a major determinant of therapy resistance in breast cancer cells. For instance, Wei et al. showed that both NRF2 and p62 were overexpressed in breast cancer samples compared to normal tissues but, also, in MCF-7/ADR breast cancer cells compared to MCF-7 cells [41]. Interestingly, the genetic silencing of either NRF2 or p62 revealed the existence of a reciprocal regulation between these two proteins and led to the impaired cell proliferation and increased sensitivity of MCF-7/ADR but not MCF-7 cells to doxorubicin. Of note, these data were also confirmed in vivo, since the administration of the anticancer drug, PA-MSHA (*Pseudomonas aeruginosa* mannose-sensitive hemagglutinin) to MCF-7/ADR-xenografted mice was able to significantly impair the tumor growth by downregulating the NRF2 and p62 levels.

A study from Del Vecchio et al. tried to elucidate the role of NRF2 in the acquisition of multidrug resistance (MDR) caused by cellular dedifferentiation by comparing normally differentiated or dedifferentiated isogenic human breast epithelial cells by the induction of epithelial-to-mesenchymal transition (EMT) due to the expression of the transcription factor TWIST [42]. Here, in differentiated cells, NRF2 activation was mediated by its oxidation, while in dedifferentiated cells, it was caused by PERK-dependent phosphorylation. These findings were further substantiated in therapy-resistant basal breast cancer cells and animal models, wherein the inhibition of the PERK–NRF2 axis reverted the MDR phenotype and sensitized the drug-resistant cancer cells to chemotherapy, decreasing their intracellular GSH content. In addition, the analysis of the patient tumor datasets revealed that the PERK gene expression signature positively correlated with a basal breast cancer gene signature, tumor grade and chemotherapy resistance while it was negatively correlated with the differentiation status and the overall survival of the patients. Based on these data, the authors proposed that dedifferentiated breast cancer cells upregulate MDR-related genes through PERK–NRF2 signaling, while targeting this pathway might increase the sensitivity of poorly differentiated tumors refractory to anticancer drugs treatment.

Another experimental work elucidated the functional interrelation between NRF2 and heat shock factor-1 (HSF1), a protein that is frequently overexpressed and implicated in the survival and proliferation of cancer cells, also correlated with a poor prognosis of cancer patients [43]. Here, by using different cancer cell lines, including MCF-7, the authors showed that NRF2 could interact with two distinct AREs sites within the HSF1 promoter, inducing a marked increase in its mRNA and protein levels in response to oxidative or proteotoxic stress. Importantly, the NRF2–HSF1 axis was found to play an important role in MCF-7 malignancy, since interfering with NRF2-mediated HSF1 activation also suppressed the survival, migration and the expression of E-cadherin and N-cadherin, two markers of EMT in MCF7 breast cancer cells. These results demonstrated that NRF2 can transcriptionally regulate HSF1 and that this event plays an important role in the progression of breast cancers, influencing the growth, migration and survival of malignant cells. The importance of NRF2 in chemoresistance of breast cancer cells was further substantiated in a recent study wherein NRF2 activation in response to hypoxia-induced ROS accumulation conferred an insensitivity to cisplatin in MCF-7 cells [44]. Here, the enhanced NRF2 nuclear accumulation was found to induce the expression of antioxidant enzymes, GCLC and GCLM, leading to increased GSH biosynthesis, under hypoxic conditions. Importantly, the genetic or pharmacologic inhibition of NRF2 activation restored the sensitivity to cisplatin in vitro, while the concomitant use of the NRF2 inhibitor trigonelline was able to potentiate the efficacy of cisplatin in a xenograft mouse model. Finally, Carlisi et al. focused on the role of NRF2 in the development of chemoresistance using a model of triple-negative breast cancer [45]. Here, prolonged incubation of MDA-MB-231 cells with sublethal doses of doxorubicin or mitoxantrone led to an increased expression of NRF2 and the acquisition of a resistant phenotype. Of note, the use of parthenolide, a sesquiterpene lactone known for its anti-inflammatory and anticancer effects, was able to partially restore the sensitivity to both doxorubicin and mitoxantrone by preventing the overexpression of NRF2 and its target proteins and promoting intracellular ROS accumulation. Taken together, these data indicate that, in most cases, NRF2 mediates a therapy resistance in breast cancer cells in response to the altered redox balance caused by anticancer drug administration, but this does not exclude the possibility that other types of mechanisms, ROS-independent, might also account for enhanced NRF2 signaling under different biological contexts.

#### 2.3.3. NRF2 in Metabolic Adaptation of Breast Cancer Cells

It is well-recognized that metabolic reprogramming is a common hallmark of cancer cells and that NRF2 participates in the regulation of metabolic pathways supporting tumor progression [46]. In a recent study, it was demonstrated that the overexpression of NRF2 can promote the proliferation and migration of breast cancer cells by upregulating the expression of glucose-6-phosphate dehydrogenase (G6PD), a key enzyme in the pentose phosphate pathway (PPP) [47]. More in detail, by using MCF-7 and MDA-MB-231 breast cancer cells, the authors showed that the inhibition of NRF2 and overexpression of Kelch-like ECH-associated protein-1 (KEAP1) reduced the expression of G6PD, while NRF2 overexpression or KEAP1 knockdown had the opposite effect. Additionally, the dissection of the molecular mechanism revealed that NRF2 promoted the expression of Notch1 through the activation of the G6PD/HIF-1α (hypoxia-inducing factor 1α) pathway in both MCF-7 and MDA-MB-231 cells. Importantly, the NRF2 genetic depletion was significantly abrogated, while its overexpression or KEAP1 knockdown markedly enhanced breast cancer cell proliferation, migration and invasion [47]. Therefore, the authors concluded that NRF2 plays an essential function in the regulation of breast cancer malignancy by influencing the Notch1 signaling pathway through the upregulation of G6PD, a rate-limiting enzyme of the PPP. Consistently, other experimental works have provided evidence supporting the notion that the NRF2-dependent modulation of HIF-1α downstream signaling might represent a conserved mechanism through which breast cancer cells undergo metabolic adaptation and reprogramming [48,49].

A study from Walker et al. showed another pathway for the metabolic adaptation of breast cancer cells [50]. According to their experimental work, a glucose deprivation in cancer cells led to the induction of autophagy, which supports cellular survival. This metabolic adaptation under the metabolic stress condition also decreased the p62 levels and eventually upregulated the NRF2 levels. The increased NRF2 levels in cancer cells also maintained the regular ROS levels. This data supports the role of NRF2 in the metabolic adaptation of cancer cells to a nutrient-deprived environment.

In another study, the analysis of human breast cancer datasets revealed that the expression of caveolin-1 (CAV-1) was inversely correlated to that of NRF2 or superoxide dismutase-2 (SOD-2), and this could predict the development of more aggressive forms of cancer [51,52,53]. Interestingly, the reconstitution of CAV-1 expression in MCF-7 breast cancer cells that are normally defective for this protein was sufficient to suppress the NRF2 activity by promoting its interaction with KEAP1 and, consequently, its faster degradation, an event ultimately leading to a decreased SOD-2 expression. Of note, these changes were sufficient to induce a metabolic switch characterized by impaired glycolytic activity and increased mitochondria-dependent ATP production. The mechanistic insights revealed that CAV-1 loss induces SOD-2 upregulation and H_2_O_2_ accumulation, which, in turn, promotes AMPK activation and, ultimately, enhances the glycolytic rate of the MCF-7 cells. Consistently, the rescued expression of CAV-1 led to a marked inhibition of anchorage-independent growth and suppressed the AMPK-dependent activation of the glycolytic switch through the inhibition of H_2_O_2_ production derived from the SOD-2-dependent conversion of the superoxide anion. Furthermore, by using a mouse model of genetically induced breast cancer, the reduced expression of CAV-1, associated with elevated SOD-2 and enhanced AMPK activation, was also confirmed in tissue sections from mammary tumors but not in their healthy counterparts. Therefore, the authors concluded that the progressive loss of CAV-1 during breast cancer progression induces the activation of NRF2 and the subsequent upregulation of SOD-2, promoting an AMPK-dependent glycolytic switch that is permissive to the acquisition of a highly aggressive phenotype that negatively impacts the overall survival and prognosis of patients [54].

#### 2.3.4. NRF2 in Breast Cancer Prognosis

Accumulating evidence indicates that NRF2 is frequently overexpressed in different types of malignant tumors and associated with a poor prognosis. However, the pathological and clinical significance of NRF2 in breast cancers has revealed contrasting results. For example, an early study showed that NRF2 was frequently depleted in breast cancer biopsies and breast cancer cell lines due to its augmented proteasomal degradation caused by the concomitant overexpression of the E3 ubiquitin ligase CUL3 [55]. Here, the use of siRNA against CUL3 in MCF-7 breast cancer cells increased the levels of NRF2-regulated proteins, including GCL, NQO1, AKR1C1, UGDH, TXN and the drug transporter ABCC1, ultimately conferring a resistance to the oxidants and conventional anticancer agents. Despite the limited number of samples analyzed, and the lack of clear correlations between NRF2 and tumor grading or the survival of patients, the authors proposed that the molecular signature characterized by a high CUL3 and low NRF2 content might identify a cohort of patients more sensitive to chemical carcinogenesis but, also, to the use of chemotherapeutic drugs.

The first study demonstrating an association between nuclear NRF2 immunoreactivity and the adverse clinical outcome of breast cancer patients was conducted by Onodera et al. [56]. Here, by analyzing around 100 specimens of invasive breast carcinoma, nuclear NRF2 immunoreactivity was detected in 44% of the carcinoma cases, while the NRF2 status was significantly associated with the NQO1 and p62 protein contents, the Ki-67 index and the histological grade. Of note, the multivariate analysis revealed that the NRF2 status was an independent adverse prognostic factor for both the recurrence and disease-free survival of the patients, while in vitro studies confirmed that NRF2 was able to control the proliferation and migration of the MCF-7 and SK-BR-3 cells. These results indicate that the nuclear NRF2 status plays an important role in controlling the progression of breast cancer and might be considered a robust prognostic marker in breast cancer patients. In another study, Hartikainen et al. investigated the significance of NRF2 expression and its target gene sulfiredoxin (SRXN1) by using tissue microarrays representative of invasive breast carcinomas [57]. Here, nine single-nucleotide polymorphisms of the NRF2 gene were analyzed in 452 patients with breast cancer and 370 controls while the subsequent protein expression analysis revealed high cytoplasmic NRF2 positivity in 66% (237 of 361) and nuclear positivity in 26% (96 of 365) of the cases. More in detail, the authors showed that the NRF2 polymorphism rs6721961 was associated with breast cancer risk, while the NRF2 rs2886162 AA genotype variant independently predicted a poorer survival among patients who received chemotherapy or radiotherapy. These data indicated that the NRF2 pathway can influence both the predisposition toward developing breast cancer and the overall clinical outcome of breast cancer patients, confirming its importance in cancer progression. In a later study from the same group, it was consistently demonstrated that genetic polymorphisms in KEAP1 also affect the breast cancer risk and clinical outcome, modifying the effects of radiotherapy and tamoxifen treatments on patient survival [58]. Interestingly, among the five genetic variants, the KEAP1 rs11085735 minor allele A was significantly associated with a lower KEAP1 protein expression and high NRF2 nuclear expression. Additionally, when the treatment data were included, a multivariate survival analysis revealed that this SNP was associated with a poorer relapse-free survival and breast cancer-specific survival among all the invasive cases and with a shorter relapse-free survival among the tamoxifen-treated cases. In conclusion, the authors confirmed that the genetic variants in the KEAP1 gene were associated with the outcomes of patients with breast cancer and that these SNPs may cause defects in the antioxidant defense mechanisms, underscoring the importance of the NRF2/KEAP1 signaling pathway.

An indirect confirmation that high levels of NRF2 adversely impact the prognosis of breast cancer patients comes from a quite recent study wherein the authors analyzed the expression data and clinical data from The Cancer Genome Atlas (TCGA) [59]. Here, by using univariate and multivariate survival analyses, it was shown that the median survival time of patients with a low SETD7 expression (18.1 years) was twice as much that of patients expressing high levels of SETD7 (9.5 years), a SET domain-containing lysine methyltransferase 7 that monomethylates histone and nonhistone proteins. Additionally, the SETD7 expression was found to positively correlate with the expression of NRF2 and a bunch of its target genes—namely, ME1, TXNRD1, GCLC and GCLM. The mechanistic insights revealed that the stable knockdown of SETD7 significantly impaired the cell proliferation and viability in MCF-7 and MDA-MB-231 breast cancer cells, also leading to an increased intracellular ROS content and a decreased GSH/GSSG ratio due to the repression of NRF2-dependent antioxidant genes expression. Therefore, the authors concluded that SETD7 is a prognostic marker in breast cancer patients and an upstream transcriptional regulator of antioxidant proteins in breast cancer cells dependent on the KEAP1-NRF2 pathway.

In another study from Lu et al., it was shown that dipeptidyl-peptidase 3 (DPP3), a KEAP1-binding protein that promotes NRF2 accumulation by competitively binding and sequestering KEAP1, was able to influence NRF2 signaling in breast cancer cells [60]. Here, MCF-7 ER (+) breast cancer cells were used to demonstrate that the DPP3 interaction with KEAP1 was dose-dependently reinforced by increasing the amount of oxidative stressors. Mechanistically, DPP3 promoted NRF2 nuclear accumulation and activity through a competitive binding with KEAP1 independently from its enzymatic activity, while its overexpression enhanced the KEAP1 levels and its genetic knockdown prevented H_2_O_2_-dependent NRF2 nuclear accumulation. Finally, by analyzing the data from the TCGA database, DPP3 was found to be overexpressed in human breast cancer and to correlate with increased NRF2 target gene expression and poor prognosis, especially in ER (+) breast cancer. Based on these data, the authors proposed that DPP3 overexpression promotes breast cancer progression, metastasis and drug resistance, stabilizing NRF2, while the signature characterized by high levels of NRF2 and DPP3 might represent a potential biomarker for breast cancer prognosis and treatment.

In a marked contrast, other evidence has shown that the NRF2 mRNA levels analyzed in two independent breast cancer patient cohorts were inversely correlated with the clinical outcome of the disease [61]. Indeed, not only the NRF2 mRNA levels were higher in normal breast tissue than in breast tumor tissue of the same patient, but patients with high NRF2 mRNA levels had a better disease-specific survival and overall survival compared to those with low NRF2 mRNA contents. Interestingly, this prognostically relevant association was even more pronounced in the subgroup of patients with ER (+), but not in patients with ER (−), tumors. These data support the notion that NRF2 can also act as a tumor suppressor in breast cancer, underlying the complexity of NRF2/KEAP1 signaling.

In summary, the available data indicate that NRF2 can have both pro- and antioncogenic effects, most likely depending on additional factors that are context-specific. Among the others, the genetic background, the subcellular location, the presence of genetic polymorphisms, the interactions with upstream and downstream regulators and the KEAP1 status represent the major determinants of whether NRF2 will exert tumor-promoting or tumor-suppressive functions. These findings underscore the intricate complexity of NRF2 signaling and pave the way to further investigations aimed at elucidating the clinical impact of NRF2 in breast cancer.

## 3. NRF2-Related Mechanisms as a Target in Breast Cancer

NRF2 activation shows important activities against oxidative stress and xenobiotic detoxification, which assure NRF2′s preventive role against cancer. However, recent studies have shown that excessive NRF2 levels in cancer can cause cell metabolism reprogramming, resulting in chemo- and radiotherapy resistance. NRF2 targeting in cancer therapy mainly relies on two different mechanisms: NRF2 activation to prevent cancer development and NRF2 inhibition to improve cancer therapy sensitivity [62]. In this section, the possible roles of natural molecules, polyphenols and melatonin in breast cancer prevention and therapy via NRF2-related mechanisms are reviewed.

### 3.1. Therapeutical Role of Polyphenols

Curcumin, which is a polyphenolic metabolite of *Curcuma* spp., induces NRF2; facilitates the upregulation of antioxidative enzymes such as NQO1, HO-1, GST and glutathione reductase (GR) and enables cellular senescence [63]. The mechanism under NRF2 activation by curcumin relies on the modulation of KEAP1 thiol [64]. A previous study from Rushworth et al. provided evidence of another mechanism, since curcumin mediates NRF2 phosphorylation via stimulating PKC and enhances the ARE-mediated expression of HO-1 and GCLM in human monocytes [65]. In a study that set out to determine the NRF2-mediated HO-1 activation by polyphenols, curcumin was shown to induce the NRF2 and HO-1 protein levels in MDA-MB-468 and HBL100 breast cancer cells [66]. The researchers also investigated wild type (NRF2 +/+) and null (NRF2-/-) mouse embryo fibroblasts and revealed that curcumin induces HO-1 through NRF2 activation. In another study, the oxidative DNA damage induced by benzo-a-pyrene (BaP), a well-known human carcinogen, was shown to be prevented by a curcumin treatment in Swiss albino rats [67]. A curcumin treatment also decreased the CYP1A catalyzed bioactivation of BaP and induced the NRF2, GST and NQO1 protein levels. In contrast with this study, Jain et al. showed that curcumin downregulated the 2-amino-1-methyl-6-phenylimidazo(4,5-b) pyridine (PhIP; the heterocyclic amin is possibly carcinogenic to humans)-induced NQO1 expression while inhibiting the DNA adduct and ROS formation in MCF-10A cells. The authors concluded that the increased NRF2 activation ensures cells reduce the oxidative stress and DNA adduct formation [68]. However, the authors did not explain that the NQO1 expression is regulated via NRF2, which has been shown to be reduced by curcumin. NRF2 regulates not only ARE but, also, Flap endonuclease I (FENI), which has a role in DNA repair, as well as cell proliferation. Curcumin inhibits MCF-7 cell proliferation while inducing the NRF2 levels in a dose-dependent manner [69]. Here, the NRF2 protein downregulated the FENI expression. Since tumor aggressiveness was found related to the increased FENI activity, this study revealed that NRF2 activation by curcumin may also be effective in cancer therapy. Curcumin’s inhibitory effect on breast cancer cell proliferation also mediated with the inhibition of oncogenic miR-19. MCF-7 cell transfection with miR-19a and -19b represented the decreased PTEN, AKT and p53 pathway-associated protein levels [70]. Moreover, curcumin inhibited bisphenol A-induced cell proliferation; downregulated miR-19a and -19b and modulated the PTEN, AKT and p53 expression. Taken together, both in vitro and in vivo studies have provided important insights into curcumin’s preventive role in chemically induced DNA damage and oxidative stress. Additionally, curcumin-mediated NRF2 activation may have an impact on cancer therapy via its downstream gene-related tumor growth inhibition.

Epigallocatechin-3-gallate (EGCG) is most abundantly found in green tea. This polyphenol induces NRF2 and downstream genes—mainly, phase II detoxification enzymes, similarly to curcumin [71]. NRF2 induction by EGCG was investigated with the Western blot analysis in MCF-7 and MDA-MB-231 breast cancer cells. According to the study, EGCG increased the NRF2 levels, and consequently, both cell lines became resistant to the growth inhibitory effects of doxorubicin or paclitaxel [72]. In contrast, the EGCG cotreatment reduced the apoptotic response of cisplatin in MDA-MB-231 cells [71]. The authors also studied a triple-negative breast cancer model in vivo with 4T1 murine breast cancer cells, showing that a cotreatment of cisplatin with EGCG suppressed tumor growth more potently than cisplatin alone. Increased oxidative stress in the cancer cells resulted in enhanced tumor growth and chemoresistance [73]. NRF2 induction by polyphenols such as curcumin and/or EGCG can cope with oxidative stress and improve the sensitivity to chemotherapy. However, these controversial findings can be explained by the fact that polyphenols also induce the NRF2 downstream phase II detoxification pathway, which metabolizes chemotherapeutics easily and causes chemoresistance.

The grapefruit polyphenol resveratrol is well-known for its biphasic effects [74]. At low concentrations, the resveratrol treatment induces cell proliferation in breast cancer cells, while its increased concentration causes cytotoxicity. Similarly, the antioxidant features of resveratrol have been seen in low concentrations, while it represents the prooxidant profile at higher dosages. A study from Rai et al. treated MCF-7 and MDA-MB-231 cells with resveratrol between the 50–400-µM concentration range, which had strong cytotoxicity in a dose-dependent manner [75]. Furthermore, in MCF-7 cells, a resveratrol combination with doxorubicin acted synergistically to inhibit cell proliferation, colony formation, cell migration and to promote apoptosis while, at the protein level, downregulate the regulatory genes of inflammation (NF-kB and COX-2), oxidative stress (NRF2) and autophagy (LC3B and Beclin-1) and increase the proapoptotic protein ratios (BAX/BCL2). Resveratrol alone increases NRF2 at the protein level. The concentration of doxorubicin was decreased to obtain a similar therapeutic efficacy with resveratrol. Resveratrol also showed a promising suppressing effect on estrogen-induced breast cancer [76]. The resveratrol treatment for DMBA-induced ER (+) breast cancer in Sprague–Dawley rats resulted with a decreased tumor volume. The study revealed that the increased expression of NRF2 with a resveratrol-treated group promoted NQO1, HO1 and UGT1A8 expression. The estradiol downregulation of NRF2 and its downstream antioxidant (NQO1 and SOD3) genes, detoxification (FMO1 and AOX1) genes and DNA repair (OGG1) gene were reinduced by resveratrol in August Copenhagen Irish rats. Similarly, an estradiol-induced mammary tumor incidence was decreased with resveratrol. In the same study, estradiol-induced DNA damage was evaluated in MCF-10A cells with both estradiol and estradiol plus resveratrol-treated groups. These studies have revealed that resveratrol induces apoptosis, decreases oxidative stress-related DNA adduct production and inhibits colony and mammosphere formation in estradiol-treated MCF-10A cells. The authors concluded that resveratrol exerted a preventive role against estradiol-induced breast tumor development via the NRF2-mediated pathway. The study was consistent with resveratrol’s protective role against BaP-induced oxidative stress in BRCA1-defective MCF-10A cells [77]. Overall, these studies confirmed that resveratrol mediated the induction of NRF2, resulting in a chemoprevention against xenobiotic-induced cancer development in contrast to the inhibition of NRF2, which resulted in chemosensitivity.

**Figure 2:**
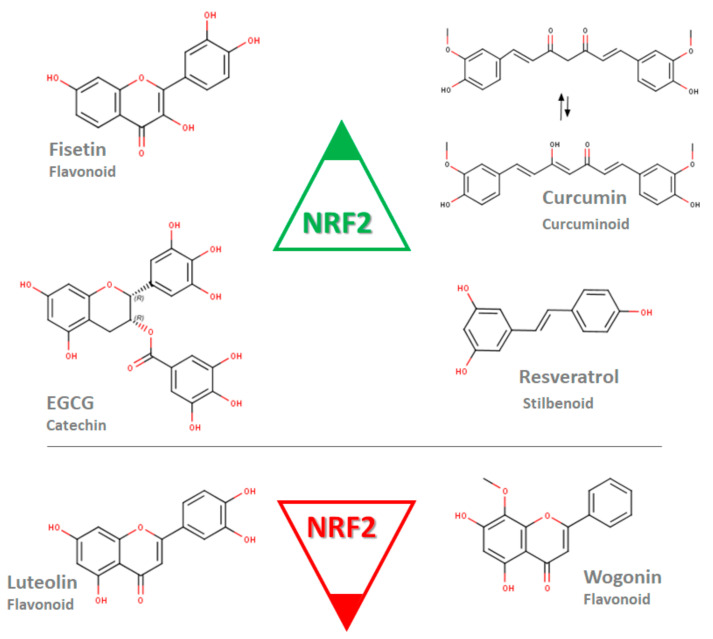
Polyphenols of various subgroups modulating the expression of NRF2 at the mRNA or/and protein level. Fisetin, epigallocatechin-3-gallate (EGCG), curcumin and resveratrol induce NRF2 in contrast to luteolin and wogonin (Marvin was used for drawing chemical structures, Marvin 20.20.0 (2020), ChemAxon [80]).

A limited mechanistical study was conducted to evaluate the inhibition of NRF2 by polyphenols. Luteolin-loaded nanoparticles with increased luteolin bioavailability were shown to decrease the NRF2, HO1 and MDR1 expression at the mRNA level in MDA-MB-231 cells [78]. Additionally, luteolin nanoparticles enhanced the doxorubicin sensitivity of MDA-MB-231 cells. A study from Zhong et al. showed that wogonin treatment remodulates increased the NRF2, HO-1 and NQO1 protein levels in doxorubicin-resistant MCF-7 cells [79]. These results suggest that the NRF2 modulation by polyphenols differs between the molecules, while fisetin, curcumin, EGCG and resveratrol induce NRF2, luteolin and wogonin and inhibit NRF2 expression (Figure 2).

### 3.2. Therapeutical Role of Melatonin

Melatonin is an indolic pineal hormone. The impaired circadian synthesis and secretion of melatonin is shown to be an important risk factor for the development and progression of breast cancer [81,82]. A plethora of research was conducted to evaluate the role of melatonin in the prevention and treatment of breast cancer, including the modulation of oxidative stress [8] and the regulation of miRNAs that are associated with apoptotic, cellular senescence and proliferation genes [83]. Melatonin also plays a crucial role in sustaining the mitochondrial functions, which was reviewed by Almeida Chuffa et al. [84]. The authors underlined that melatonin favors the mitochondria by protecting the mitochondrial proteins and mtDNA against oxidative damage and promoting electron chain activity against mitochondrial dysfunction in various cancers, including breast cancer.

**Figure 3:**
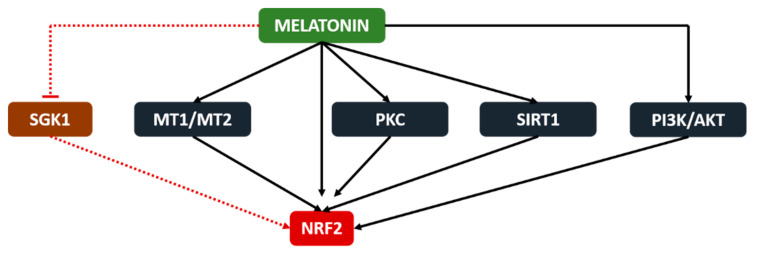
Melatonin stimulates NRF2 via the melatonin receptors (MT1 and MT2), SIRT1 and PI3K/AKT pathways in nontumorigenic cells while inhibiting the SGK1-mediated upregulation of NRF2 in tumorigenic cells.

Melatonin activates NRF2 via the upregulation of cellular mediators such as PKC [85], SIRT1 [86] and PI3K/AKT [87], according to the oxidative stress-mediated cellular responses. Furthermore, Janjetovic et al. showed that the melatonin-mediated activation of NRF2 protects melanocytes against ultraviolet light B-induced oxidative stress and DNA adduct formation [88]. Increased protein levels of NRF2, NQO1 and HO-1 were also found in a melatonin-treated colon cancer model [89]. The authors showed that melatonin reverses the downregulation of inflammation (NF-kB, STAT3 and COX2) and oxidative stress (NRF2, NQO1 and HO-1) regulatory proteins by 1, 2-dimethylhydrazine dihydrochloride, a colon cancer inducer, consequently leading to reduced inflammatory (MPO, IL-17, IL-6 and TNF-a) and oxidative stress (TBARS and GSH) responses while protecting against DNA damage. Another study confirmed the NRF2 activation by melatonin, which is mediated by melatonin receptors and resulted with increased HO-1, NQO1 and GCLC levels [90]. Contrarily, melatonin is reported to inhibit the serum and glucocorticoid-induced kinase 1 (SGK1)-mediated NRF2 upregulation in cervical cancer cells. As a result, the increased oxidative stress caused cellular damage and inhibited the tumor growth in the cervical cancer model [91]. According to these current findings, melatonin seems to controversially modulate NRF2 in tumorigenic and nontumorigenic cells, which is possibly related to the different mitochondrial functions and ROS levels in the two cell types (Figure 3).

A study from Paroni et al. evaluated melatonin’s NRF2-mediated inhibition of prostate cancer in vivo. Here, melatonin induced NRF2, HIF1a and the VEGF protein while reducing the microvessel occurrence and CD31, Ki67-positive tumor cell number and inhibited tumor growth [92]. Melatonin was reviewed extensively as a “full-service anticancer agent against breast cancer” in a recent paper [93]. However, the NRF2-mediated actions of melatonin in breast cancer development and therapy have not been investigated in detail yet.

## 4. Conclusions

Oxidative stress-related mechanisms are known to be involved in the initiation, promotion and progression stages of breast cancer. NRF2 as an antioxidant response transcription factor might have a role in cancer prevention and cancer treatment. In the present review, both polyphenols and melatonin, well-known “oxidative stress modulators”, are reported to have therapeutic potential in breast cancer via the activation of NRF2. Future mechanistic studies will shed light on the NRF2-related potential of melatonin in inhibiting breast cancer initiation, promotion and/or progression.
